# Application of Multiple-Locus Variable Number Tandem Repeat Analysis to Identify Outbreak-Associated *Neisseria meningitides* Serogroup C Sequence Type 4821 in China

**DOI:** 10.1371/journal.pone.0116422

**Published:** 2015-01-20

**Authors:** Xiaoying Shan, Haijian Zhou, Ji Zhang, Bingqing Zhu, Li Xu, Guangchun Hu, Aiying Bai, Zhujun Shao, Baofa Jiang

**Affiliations:** 1 Department of Epidemiology and Health Statistics, School of Public Health, Shandong University, Jinan City, Shandong Province, P.R. China; 2 State Key Laboratory for Infectious Disease Prevention and Control, National Institute for Communicable Disease Control and Prevention, Chinese Center for Disease Control and Prevention, Beijing, P.R. China; 3 Jinan Municipal Center for Disease Control and Prevention, Jinan City, Shandong Province, P.R. China; 4 Collaborative Innovation Center for Diagnosis and Treatment of Infectious Diseases, Hangzhou City, Zhejiang Province, P.R. China; Georgia Institute of Technology, UNITED STATES

## Abstract

*Neisseria meningitidis (N. meningitidis)* serogroup C sequence type (ST)-4821 caused an outbreak in 2010 in Shandong province of China. Twenty-one non-outbreak-associated strains were isolated, along with twenty-eight *N. meningitides* serogroup C ST-4821 isolates. Therefore, it’s essential to identify and clarify characterization of the real outbreak-associated strains with a rapid method during an outbreak investigation. In this study, multiple-locus variable number tandem repeat analysis (MLVA) was applied to analyze 84 *N. meningitidis* strains, among which 58 were recovered from two outbreaks and 26 were sporadic isolates. Three MLVA schemes with different combination of VNTR loci were tested, and two of them were suitable for isolates from China: scheme 2 with six loci was found to separate ST into finer resolution, and scheme 3 with five loci can be used to identify outbreak-associated isolates from the same outbreak that caused by *N. meningitidis* serogroup C ST-4821.

## INTRODUCTION

*Neisseria meningitidis (N. meningitidis)*, also known as meningococcus, is a Gram-negative bacterium that is exclusively adapted to humans, and its natural habitat is the nasopharynx. This organism causes meningitis and other forms of meningococcal disease, such as meningococcemia, and remains a major worldwide health problem [1]. Not only is it a major cause of morbidity and mortality during childhood in industrialized countries, but also has been responsible for epidemics in Africa and in Asia [2]. Based on the immunogenicity and structure of the capsule polysaccharide, *N. meningitidis* is classified into 12 serogroups, among which serogroup A, B, C, W and Y were major epidemic and caused most meningococcal diseases [3]. From the 1950s to the 1980s, more than 95% of meningococcal cases in China were caused by *N. meningitidis* serogroup A, while serogroups B and C were less common and caused only sporadic [4]. During the following two decades, only sporadic cases were reported. However, in 2003, an outbreak caused by *N. meningitidis* serogroup C occurred in Anhui province of China, and then other six outbreaks were reported between 2003 and 2005 [5]. All these *N. meningitidis* serogroup C isolates that caused outbreaks above belong to sequence type (ST) 4821 by multilocus sequence typing (MLST), which groups meningococci according to the allelic profile of seven housekeeping genes, thus assigning them into ST clonal complexes [6]. The majority of cases of invasive meningococcal disease were caused by *N. meningitidis* which belong to a limited number of clonal complexes known as the hypervirulent lineages (including the ST-1, ST-4, ST-5, ST-8, ST-11, ST-32, ST-41/44 and ST-269 clonal complexes) [7]. ST-4821 complex is a new hypervirulent lineage which was first identified in China in 2003, then meningococcal polysaccharide vaccines A and C were used for routine immunization since then [5]. In the following five years, increased numbers of *N. meningitidis* serogroup C strains have been identified but only caused sporadic cases. However, in 2010, they caused an outbreak again in China [8]. Although this outbreak was caused by *N. meningitidis* ST-4821 serogroup C, 21 non-outbreak-associated strains that belong to other serogroups or STs were isolated at the same time. The real outbreak associated clone should be confirmed when outbreak occurred, while it cannot be separated from sporadic isolates only by serogroup typing. Molecular typing methods including pulsed-field gel electrophoresis (PFGE) and MLST have been developed and used for identifying outbreaks of meningococcal disease, however, both of them have weaknesses: PFGE is labor intensive and costly, and the interlaboratory comparison of data is challenging; while MLST is unfeasible in clinical laboratories as routine application because it is not only costly but also takes several days for sequence typing [9, 10]. Therefore, another molecular typing method, multiple-locus variable number tandem repeat analysis (MLVA), was taken in the present study to identify outbreak-associated isolates in two outbreaks caused by *N. meningitidis* serogroup C ST-4821 in China.

## MATERIALS AND METHODS

### Ethics statement

This study was approved by the scientific and ethics committees of the National Institute for Communicable Disease Control and Prevention, Chinese Center for Disease Control and Prevention. All of the participants enrolled in this study belonged to the national surveillance system of meningococcal disease. For the healthy carriers, written ethics statements were completed by the participants. All bacterial specimens isolated from patients were collected for diagnostic testing in hospitals at the request of the attending doctors; the bacteria isolated in hospitals were transported to the Chinese CDC for further research, as mandated by Chinese legislation. The consent of the patients for the diagnostic testing of specimens, including bacterial culture, was verbally attained by doctors in hospitals. The medical records were considered legal documents.

### Meningococcal Isolates

Eighty-four *N. meningitidis* strains isolated from invasive meningococcal patients or their close contacts were used in this study. These isolates comprised 26 sporadic strains from thirteen provinces between 2004 and 2012 and 58 strains from two outbreaks: 49 were isolated from outbreak 1 which occurred in Shandong province in 2010, and 9 from outbreak 2 which occurred in Anhui province in 2003 (Table 1) [5,8]. In outbreak 1, twenty-eight strains of ST-4821 were considered as outbreak-associated isolates, and 21 strains of other STs were considered as non-outbreak-associated isolates. In outbreak 2, eight strains of ST-4821 were considered as outbreak-associated isolates, and one strain of ST-2146 were considered as non-outbreak-associated isolates. All isolates were cultured on Columbia blood plate at 37°C in a 5% CO_2_ atmosphere overnight, and identified via Gram-staining, oxidase reaction and biochemical tests. DNA was extracted using a nucleic acid extraction kit (QIAGEN) following the manufacturer’s instructions. Serogroups were identified via slide agglutination using polyclonal antiserum (Difco, Fisher Scientific, Paris, France) and serogroup-specific PCR.

**Table 1:** Characteristics of the *N. meningitidis* strains used in this study.

**Strain Numbers**	**PFGE Pattern**	**Sequence Type**	**Serogroup**		**Year of Isolation**	Geographic*	**Source Type**
			**Agglutination**	**PCR**			
Strains from the outbreak 1[8]							
18	NMNh.CN0244	ST-4821	C	C	2010	1	Patient or Contact
3	NMNh.CN0001	ST-4821	NG	C	2010	1	Contact
6	NMNh.CN0244	ST-4821	NG	C	2010	1	Contact
1	NMNh.CN0244	ST-4821	NG	Other	2010	1	Contact
1	NMNh.CN0259	ST-2146	NG	A	2010	1	Contact
1	NMNh.CN0253	ST-32	NG	C	2010	1	Contact
1	NMNh.CN0261	ST-3200	B	B	2010	1	Contact
1	NMNh.CN0245	ST-5586	NG	Other	2010	1	Contact
1	NMNh.CN0256	ST-5662	NG	B	2010	1	Contact
1	NMNh.CN0251	ST-5810	NG	Other	2010	1	Contact
2	NMNh.CN0250	ST-5819	NG	B	2010	1	Contact
1	NMNh.CN0255	ST-5855	NG	Other	2010	1	Contact
1	NMNh.CN0258	ST-6621	NG	B	2010	1	Contact
1	NMNh.CN0252	ST-6927	NG	B	2010	1	Contact
1	NMNh.CN0257	ST-8489	C	C	2010	1	Contact
1	NMNh.CN0247	ST-8490	NG	B	2010	1	Contact
1	NMNh.CN0247	ST-8490	B	B	2010	1	Contact
1	NMNh.CN0179	ST-8491	W135	W135	2010	1	Contact
1	NMNh.CN0260	ST-8492	C	C	2010	1	Contact
1	NMNh.CN0246	ST-8493	B	B	2010	1	Contact
1	NMNh.CN0254	ST-92	NG	Y	2010	1	Contact
1	NMNh.CN0248	ST-9236	NG	A	2010	1	Contact
1	NMNh.CN0262	ST-9237	NG	B	2010	1	Contact
1	NMNh.CN0249	ST-9251	NG	Other	2010	1	Contact
Strains from the outbreak 2 [5]							
8	NMNh.CN0001	ST-4821	C	C	2003	2	Contact
1	NMNh.CN0013	ST-2146	NG	NG	2003	2	Contact
Strains from sporadic							
12	NMNh.CN0001	ST-4821	C	C	2004–2007	2,3,10,11,12,13	Patient or Contact
1	NMNh.CN0002	ST-4821	C	C	2006	1,2	Patient
1	NMNh.CN0004	ST-4821	C	C	2007	2	Patient
1	NMNh.CN0006	ST-4821	C	C	2005	4	Patient
1	NMNh.CN0040	ST-4821	C	C	2006	1	Patient
9	-	ST-4821	C	C	2008–2012	2,5,6,7,8,9,10	Patient

-: PFGE weren’t performed.

*:1-Shandong, 2-Anhui, 3-Beijing, 4-Fujian, 5- Gansu, 6- Guangdong, 7- Hebei, 8- Hubei, 9-Hunan, 10-Jiangsu, 11-Jiangxi, 12-Jilin, 13-Shanghai.

### MLVA

In this study, MLVA was performed using sixteen VNTR loci which were reported in previous studies (Table 2) [11–13]. Primers were same as described in these references. The sample preparation was as following: the multiplex reactions (total volume: 25 μL) contained 2.5 μL 10×buffer, 2 μL dNTP mix (2.5 mM), 1 μL DNA template, and 0.1μL Taq polymerase (500 U/μL), and the final primer concentrations were 0.4μM. The initial PCR consisted of a preheating at 95°C for 5 min, followed by 30 cycles of 95°C for 30 s, 56°C for 30 s and 72°C for 45 s, and a final incubation at 72°C for 7 min. The PCR products were analyzed by capillary separation (GeneScan ROX-500 size standard, PE Applied Biosystems) on a PE Applied Biosystems ABI Prism 3730 instrument.

**Table 2:** Repeat Sequences and Primer Sequences Used for MLVA of *N. meningitides.*

**VNTR LOCI**	**Repeat Sequence**	**Primer**
VNTR1	CAAACAA	same to NMTR1 [13]
VNTR2	CATTTCT	same to NMTR2 [13]
VNTR3	GCTTCAGTTACAGCTTCTTTG	same to NMTR6 [13]
VNTR4	CAAG	same to NMTR7 [13]
VNTR5	GCCAAAGTT	same to NMTR9 [13]
VNTR6	CCGCTGCTACTGCCGCTGCTGAAGCACCTG	same to NMTR9a [13]
VNTR7	TACGGCTGCCGCGTCAAA	same to NMTR9b [13]
VNTR8	CGGATACGCTCTTGG	same to NMTR9c [13]
VNTR9	CAGATT	same to NMTR10 [13]
VNTR11	GGGTAGCGG	same to NMTR18 [13]
VNTR12	CGTATTTTCCCAT	same to NMTR19 [13]
VNTR13	TTTCCTG	same to VNTR7-2 [11]
VNTR14	TGTTTTC	same to VNTR7-1 [11]
VNTR15	GGC	same to VNTR3-2 [11]
VNTR18	AGCC	same to VNTR4-2 [11]
VNTR19	GCTT	same to VNTR4-3 [11]

### Data Analysis

Each locus for *N. meningitidis* isolates was assigned an allele score based on the fragment size. The allele scores were converted into repeats numbers of the sixteen loci and entered into MLVA profiles, and then logged in BioNumerics version 5.1 software (Applied Maths, Kortrijk, Belgium) as character data for cluster analysis. Cluster analysis was based on the categorical coefficient and unweighted pair group method using arithmetic averages (UPGMA) method.

For the measurement of allelic diversity at each VNTR locus, Nei’s diversity index (*D*) was available in the website of HAP (http://www.hpa-bioinformatics.org.uk/cgi-bin/DICI/DICI.pl). Simpson’s diversity index (*DI*) was calculated to evaluate the discriminatory power of subtyping methods as described in previous studies [14]. The formula was as follow: *DI* = 1 - ∑ [*nj* (*nj* - 1)]/[*N*(*N* - 1)], where *nj* is the number of strains belonging to the *j*th pattern, and *N* is the number of strains in the population.

## RESULTS

### Brief description

**Figure 1:**
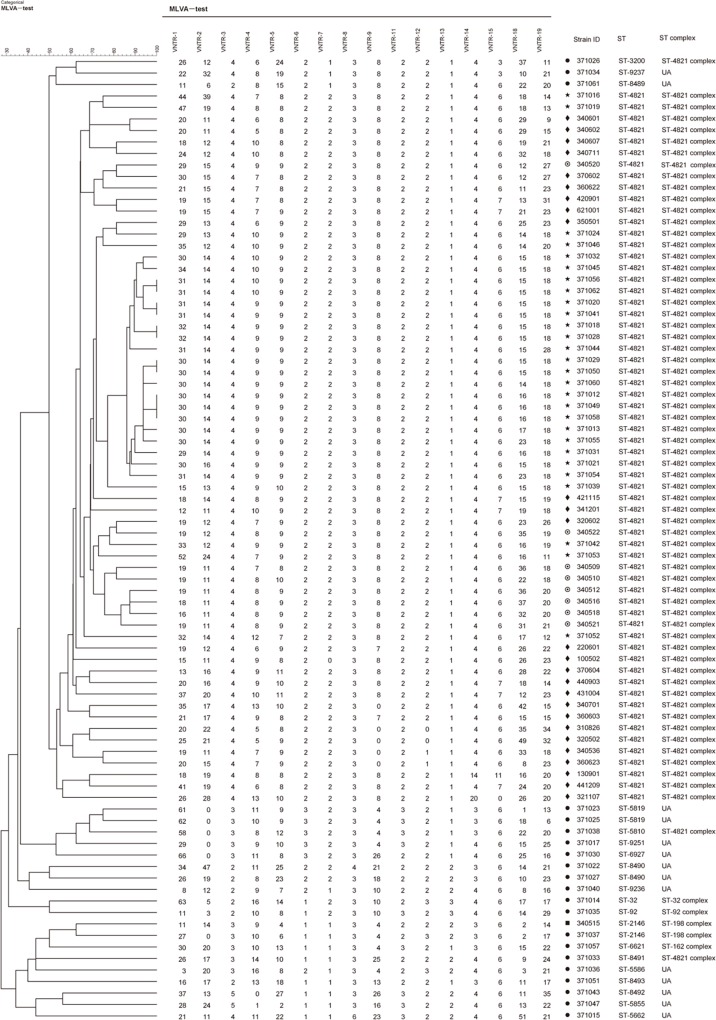
MLVA results of 84 *Neisseria meningitidis* isolates. ★ represents outbreak-associated isolates in outbreak 1; ● represents non-outbreak-associated isolates in outbreak 1; ⊙ represents outbreak-associated isolates in outbreak 2; ■ represents non-outbreak-associated isolates in outbreak 2; and ◆ represents sporadic isolates.

Sixteen VNTR loci were employed to test the number of repeats in the 84 isolates, and a total of 78 genotypes were formed (Fig. 1). Each isolate displayed a different genotype except eleven isolates from outbreak 1. Initially, characteristics of each VNTR locus in seventy-five isolates including 49 from outbreak 1 and 26 sporadic ones were demonstrated in order to identify the polymorphism (Table 3). Six loci (VNTR1, 2, 4, 5, 18 and 19) demonstrated the highest discriminatory power, while no allele variation was observed in 28 outbreak- associated isolates from outbreak 1 at other ten loci (VNTR3, 6, 7, 8, 9, 11, 12, 13, 14 and 15). Among these ten loci, five (VNTR7, 9, 12, 14, and 15) also displayed no allele variation in all 54 ST-4821 strains including 28 from outbreak 1 and 26 sporadic isolates. Based on the discriminatory power of each locus, three MLVA schemes were established: scheme1, named as MLVA4, includes four loci (VNTR4, 5, 18 and 19) which used as HV-MLVA by Schouls LM et al. [11]; scheme 2, named as MLVA6, includes the other two loci (VNTR1 and 2) besides those in scheme 1; and scheme 3, named as MLVA5, includes five lower discriminatory loci (VNTR7, 9, 12, 14 and 15) (Table 4).

**Table 3:** Main characteristics of the selected VNTR loci in *N. meningitidis* strains.

**VNTR LOCI**	**49 Strains from outbreak 1**				**28 outbreak-associated strains from outbreak 1 and 26 sporadic strains**	
	**No. of alleles**	**Nei’s diversity index (95%CI)**	**No. of alleles**	**Nei’s diversity index (95%CI)**	**No. of alleles**	**Nei’s diversity index (95%CI)**
VNTR1	11	0.8307(0.7301–0.9313)	18	0.9784(0.9504–1.0063)	22	0.9287(0.8929–0.9645)
VNTR2	7	0.4921(0.2663–0.7178)	14	0.9221(0.8406–1.0035)	14	0.8218(0.7358–0.9078)
VNTR3	1	0	4	0.7229(0.6319–0.8140)	1	0
VNTR4	5	0.5529(0.3708–0.7350)	10	0.9048(0.8566–0.9530)	8	0.7729(0.6938–0.8520)
VNTR5	3	0.2037(0.0088–0.3986)	18	0.9740(0.9395–1.0086)	6	0.5625(0.4464–0.6787)
VNTR6	1	0	3	0.6797(0.6198–0.7395)	1	0
VNTR7	1	0	2	0.5195(0.4809–0.5581)	2	0.0370(−0.0336–0.1077)
VNTR8	1	0	3	0.1775(−0.0342–0.3892)	1	0
VNTR09	1	0	10	0.8658(0.7726–0.9590)	3	0.2369(0.0917–0.3821)
VNTR11	1	0	2	0.5065(0.4003–0.5827)	1	0
VNTR12	1	0	2	0.2468(0.0339–0.4596)	3	0.1426(0.0158–0.2693)
VNTR13	1	0	3	0.6017(0.5043–0.6992)	1	0
VNTR14	1	0	2	0.4848(0.3730–0.5967)	3	0.0734(−0.0237–0.1705)
VNTR15	1	0	2	0.1732(−0.0274–0.3737)	4	0.2935(0.1454–0.4416)
VNTR18	6	0.7381(0.6090–0.8672)	17	0.9784(0.9591–0.9977)	21	0.8987(0.8419–0.9555)
VNTR19	8	0.4444(0.2106–0.6782)	14	0.9481(0.9045–0.9916)	18	0.7883(0.6811–0.8954)

**Table 4:** Three MLVA Schemes used in this Study.

**MLVA Scheme**	**Name**	**VNTR Loci used**	**Suggested usage**
Scheme 1	MLVA 4	VNTR4, 5, 18, 19	Meningococcal outbreak investigation[11]
Scheme 2	MLVA 6	VNTR1,2,4,5,18,19	Analysis of meningococcal genetic diversity and microevolution
Scheme 3	MLVA 5	VNTR7,9,12,14,15	Rapid outbreak identification of *N. meningitidis* serogroup C ST-4821

### Comparison of the MLVA results based on three schemes

**Figure 2:**
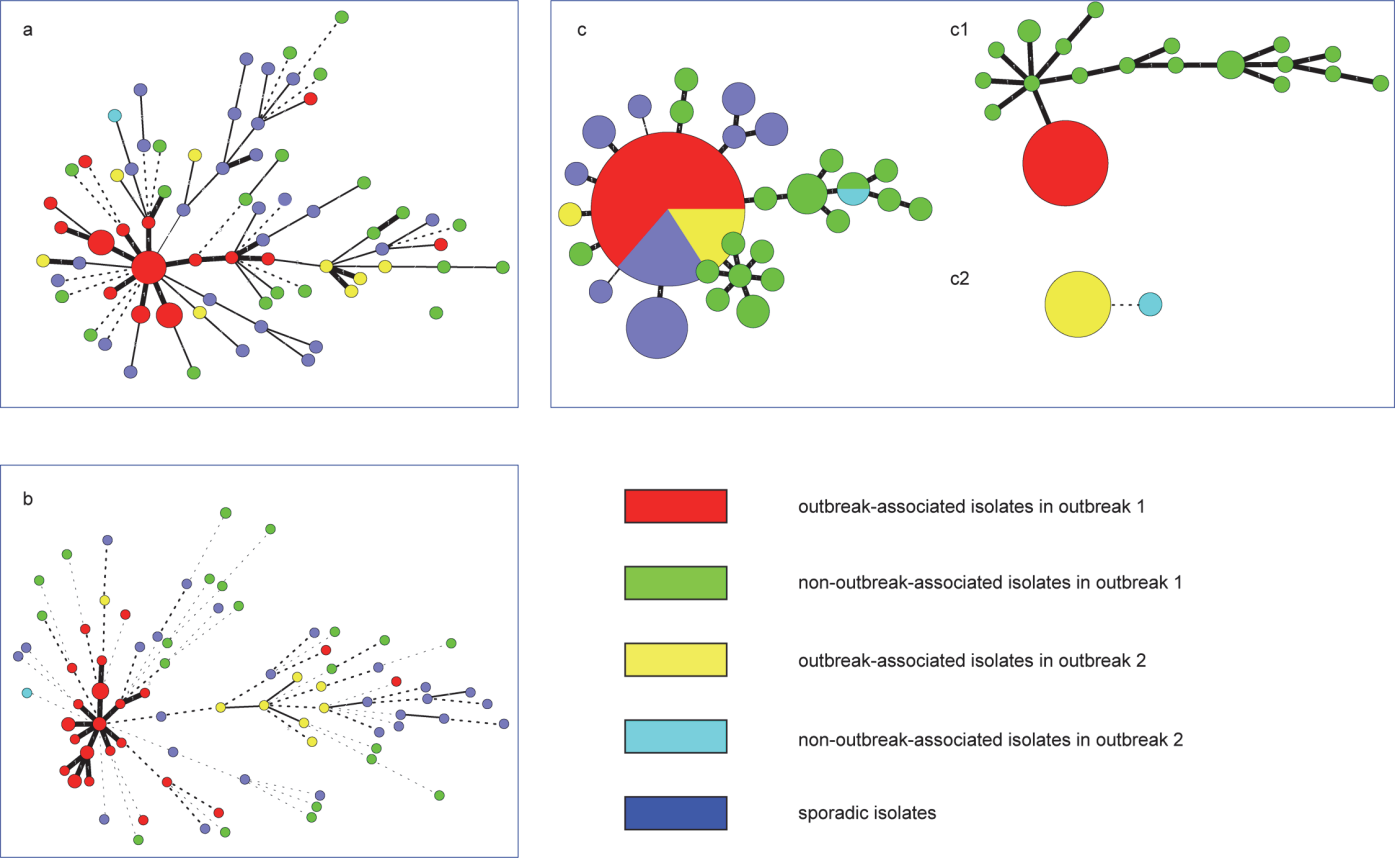
Minimum spanning trees of 84 *Neisseria meningitidis* isolates based on MLVA results. In the Minimum spanning tree (MST), each circle denotes a particular subtype by MLVA, and the size of each circle indicates the number of isolates of that particular subtype. Thick, solid lines represent single-locus variants; thin, solid lines represent double-locus variants; thick, dotted lines represent triple-locus variants; and thin, dotted lines represent quadruple or more locus variants. Five resources of strains were displayed with different colors. The relationships of 84 strains were displayed by three MLVA schemes with different combinations of VNTR loci: (a) scheme 1, named as MLVA4, with four loci (VNTR4, 5, 18 and 19); (b) scheme 2, named as MLVA6, with six loci (VNTR1, 2, 4, 5, 18 and 19); and (c) scheme 3, named as MLVA5, with five loci (VNTR7, 9, 12, 14 and 15). Relationships of isolates from outbreak 1 and outbreak 2 were displayed in c1 and c2 respectively.

Eighty-four isolates were divided into 71 genotypes by scheme 1(Fig. 2A), while they were divided into 78 genotypes by scheme 2 (Fig. 2B) and 28 genotypes by scheme 3(Fig. 2C) respectively. With scheme 1 and scheme 2, all isolates used in this study displayed polymorphism diversity, but outbreak-associated strains cannot be separated from sporadic associated ones. With scheme 3, *N. meningitidis* serogroup C ST-4821 strains displayed low polymorphism diversity and 44 isolates (including 28 from outbreak 1, 7 from outbreak 2, and 9 sporadic isolates) clustered in one group (Fig. 2C). However, when scheme 3 was employed on one outbreak, the real outbreak-associated isolates can be identified from the sporadic ones: 28 ST-4821 isolates can be identified from 21 non-outbreak-associated ones during outbreak1 (Fig. 1C1); and 8 ST-4821 isolates can be identified from one non-outbreak-associated ones in outbreak 2 (Fig. 1C2).

## DISCUSSION

*N. meningitidis* serogroup C ST-4821 was first identified in 2003 and still the major ST in China. It has been isolated in many provinces and carriage rate in healthy population can reach to 10% in some areas in China [15]. Outbreak 1 discussed in this study was reported by reported by Zhang Ji, et al. in 2013; these authors determined that the outbreak was due to a ST-4821 serogroup C *N. meningitidis*, but PFGE results showed that NMNh.CN0244 and NMNh.CN0001 were the major pathogenic types. To more accurately define the molecular type, MLVA with 16 loci was used in the present study to distinguish the *N. meningitidis* isolates collected during this outbreak and from sporadic cases between 2004 and 2012. On the base of analysis of diversity, three MLVA schemes were employed to identify outbreak-associated isolates. Scheme 1 in this study was the HV-MLVA that reported by Schouls et al. for outbreak analysis [11]. However, it is not suitable for *N. meningitidis* serogroup C ST-4821 because it cannot separate the real outbreak-associated isolates and diversity index of those four loci is lower than VNTR1 and VNTR2 used in this study. Additionally, scheme 2 with 6-locus MLVA was found to be the most effective MLVA method because it performed similarly to the sixteen loci while using fewer VNTR loci. Thus, MLVA6 can be used to determine the epidemic status of *N. meningitidis*. However, both scheme 1 and scheme 2 cannot be used to determine the real outbreak-associated *N. meningitidis* strains in one outbreak because the similar isolates will not be grouped together. In this case, scheme 3 can be used because it can cluster the outbreak-associated isolates in one group, resulting in the identification of the suspected pathogen during the same outbreak. Therefore, we can use scheme 3 to determine the main type that causes an outbreak and use scheme 2 to distinguish the molecular and epidemiological characteristics of *N. meningitidis* serogroup C ST-4821. Additionally, when we cannot obtain strains but throat swab, blood or CSF cultures during an outbreak, characteristics of pathogen based on DNA extracts can be analyzed by MLVA due to its speed, relatively simple data analysis, and considerably lower costs.
